# The Assessment of the Motor and Non-Motor Aspects of Anosognosia for Hemiplegia: A Historical Review

**DOI:** 10.3390/brainsci15040404

**Published:** 2025-04-17

**Authors:** Maddalena Beccherle, Sara Bertagnoli, Valentina Moro

**Affiliations:** 1 NPSY-Lab.VR, Department of Human Sciences, University of Verona, Lungadige Porta Vittoria, 17, 37129 Verona, Italy; maddalena.beccherle@univr.it; 2 Social and Cognitive Neuroscience Laboratory, Department of Psychology, University La Sapienza, 00185 Roma, Italy; sara.bertagnoli@uniroma1.it

**Keywords:** anosognosia for hemiplegia, motor awareness, dimensions of anosognosia, emotions in motor awareness, differential diagnosis in anosognosia

## Abstract

Anosognosia for hemiplegia is a complex, multifaceted phenomenon. Due to the various different forms in which it manifests and the few tools available to treat it, it can create difficulties for both clinicians and researchers. Since the first definition established by Babinski, a great deal of research has been performed and has shown that this deficit in motor awareness involves not only motor aspects but also other, non-motor dimensions. These dimensions all need to be taken into consideration during the process of diagnosis, in particular when planning a rehabilitation programme. This article reviews the main instruments currently available for the diagnosis of anosognosia for hemiplegia. After a description of the best tests to assess motor dimensions (such as explicit and implicit anosognosia, emergent awareness and awareness for daily life activities), non-motor dimensions are analysed. The literature on the subject provides ideas and tools for the evaluation of cognitive (i.e., motor imagery), emotional and social (i.e., theory of mind) aspects. Finally, the importance of differential diagnoses in relation to disorders often associated with anosognosia is discussed.

## 1. Introduction

Anosognosia (from the Greek language, where a = without, nose = illness and gnosis = knowledge) is defined as the lack of, or a reduction in, awareness in a patient of his or her own disorder [1,2]. In this context, awareness is considered as “a reasonable or realistic perception or appraisal of a given aspect of one’s situation, functioning or performance, or of the resulting implications, which may be expressed explicitly or implicitly” [3] (pp. 396). Anosognosia may comprise a variety of neurological cognitive deficits, such as amnesia [4,5], apraxia [6], blindness [7], symptoms of Parkinson’s disease [8] and issues relating to social abilities [9,10]. In the context of this paper, the disorder in question is hemiplegia, that is, the paralysis of one side of the body. Patients suffering from anosognosia for hemiplegia (AHP) declare that they are able to move their contralesional body parts, for example, to raise their arm, grasp an object, stand, or even walk and climb stairs. Even when faced with their failure to execute these actions, they often deny that they are suffering from this disorder or claim that they have made movements when, in fact, they have not. Nevertheless, as we will discuss below, although AHP relates to a motor deficit, awareness turns out to be a complex, multifaceted phenomenon, which also involves non-motor dimensions.

AHP is traditionally described as a consequence of right hemisphere damage, with a prevalence ranging from 15% [11] to 54.1% [12], depending on the methods used for assessment. However, when assessed by means of non-verbal tasks (e.g., asking the patient to evaluate their own ability to perform bilateral actions without asking them to actually perform the action [13]), left hemisphere-damaged patients may also show awareness disorders [14].

There are various factors that complicate the diagnostic process. Firstly, the interval between lesion onset and assessment is crucial, as AHP, which is relatively frequent in acute phases, tends to attenuate or resolve itself (therefore becoming more difficult to identify) within a few weeks from brain damage [15,16]. Furthermore, AHP is not a “consistent” clinical condition; the symptoms fluctuate, meaning that a patient gives appropriate answers at certain moments then falls into a state of unawareness a few moments later. In addition, responses are often incongruent and inconsistent, with patients who may recognise that they find it difficult to move their contralesional hand but, at the same time, declare that they are able to perform daily life activities without any problems.

AHP is also variable in its manifestation. Dissociations have been found between deficits in verbal, explicit versus implicit awareness, namely, situations in which patients who verbally deny their paralysis act as if they know they cannot move the paralysed body part (e.g., their arm) [16,17].

When asked to actually perform an action, hemiplegic patients may react in different ways: Aware patients show anticipatory awareness; that is, they are immediately aware that it is impossible for them to perform the action even when they are not being asked to execute it, while the behaviour of AHP patients depends on the severity of their awareness deficit. Indeed, some AHP patients may show emergent awareness; that is, they only become aware of their difficulties when they actually have to perform an action. Other AHP patients only demonstrate intellectual awareness; that is, they have a generic ability to recognise that they have suffered a stroke and are suffering from the potential related deficits, but even when faced with their own failures, they continue to declare that they are able to move and perform actions [18,19]. Each of these conditions requires different clinical and rehabilitation approaches [20,21,22].

Finally, AHP may be inconsistent depending on body localisation, with some patients aware of leg but not upper limb deficits, while the opposite is rarer.

Over time, the heterogeneity characterising AHP manifestations has generated a number of different interpretations of the syndrome (see for example [23,24,25,26]). Nowadays, there is a general agreement that AHP is a multifaceted syndrome resulting from damage to a complex network rather than single structures. It does not only involve the motor monitoring system (i.e., the premotor loop [27]) or the insula [28] but also affects the limbic system, as well as the ventral attentional network [19,29]. Direct and indirect damage to this “system of systems” may determine partially different manifestations, despite the fact that they share common fundamental symptoms, as represented by the lack of awareness of motor deficits [19].

In the following sections, various different approaches for the assessment of AHP will be discussed, giving specific attention to the complexity in characterising the syndrome. The main aim of this article is not to provide a systematic review of AHP, but rather to discuss the evolution of the definition of the syndrome and, thus, the procedures used for its assessment. Therefore, we cannot exclude the possibility that some aspects might be missed.

In the first section, the assessment of AHP as it relates directly to motor behaviour will be discussed. The following section will then address the non-motor dimensions of AHP and the instruments available to investigate these dimensions. Finally, in the last part of the paper, the challenges associated with differential diagnosis with respect to other cognitive disorders will be addressed.

## 2. Assessing the Motor Dimensions of AHP

The first systematic definition of AHP was published around 40 years ago in a seminal paper by Bisiach et al. [30]. This was based on a previous interview by Cutting [31], who was the first to introduce a scoring system to identify the severity of AHP symptoms. By means of this tool, patients are interviewed about their clinical conditions, and a score is given using a 4-point scale according to their responses. If the disorder is spontaneously reported by the patient following a general question about their complaints, the score is ‘0’ (i.e., no anosognosia); a score of 1 indicates that the disorder is reported following a specific question about the strength of the patient’s limbs; a score of 2 is assigned if the disorder is acknowledged only after its demonstration by means of the routine techniques used in neurological examinations, and finally, a score of 3 is given if no acknowledgement of the disorder can be obtained from the patient. Ten years later, Berti and colleagues updated this scale [32] with a more structured interview. Explicit awareness is investigated by means of a series of initial questions (i.e., Where are we? Why are you in the hospital? How is your left arm? Can you move it? Why can you not move your left arm?). Similar questions are asked for the lower left limbs. Furthermore, if patients verbally deny motor impairment in their upper left limb, they are asked to try to touch the examiner’s hand with their left hand and subsequently to state whether or not they have performed it (confrontation task). Then, an estimation of the patients’ current abilities to perform various different actions is requested (from 0 = very badly to 10 = very well).

These scales, along with other less structured clinical interviews [33,34], are very useful, especially in acute phases after lesion onset, as they are easy to administer when patients are bedridden and do not require specific tools. However, repeated administration of the interviews may lead to a sort of learning effect, that is, the risk that the patients learn the expected responses, consequently generating “false negative” results. Furthermore, the scoring system may be ambiguous, as even aware patients might not mention their paralysis spontaneously, for example, because they consider other deficits to be more prominent or important (e.g., pain), or they might report some motor impairments without relating them to the presence of hemiplegia (e.g., a previous unrelated surgical operation or arthrosis). In these cases, when patients report a motor deficit without mentioning their paralysis, the attribution of an intermediate score (1.5) has been suggested [22,35].

Over time, more structured questionnaires have been devised, which permit a quantification of the gradient of awareness. The anosognosia for hemiplegia questionnaire [36] comprises a series of 10 questions concerning the patient’s paralysed arm in terms of motricity and sensory functions. Each response is independently scored (0 = the patient shows awareness of the deficit, 0.5 = partial awareness, and 1.0 = complete unawareness or denial). Moreover, this tool introduces a new aspect of awareness regarding the patient’s ability to consider another people’s point of view (a third-person perspective), which is investigated by means of a specific question (“The doctors tell me that there is some paralysis in your arm. Do you agree?”).

A breakthrough in the assessment of the motor aspects of AHP was achieved with the introduction of non-verbal, validated evaluation tools. The VATA-M (Visual Analogue Test for Anosognosia for motor impairment, [14]) consists of two sub-scales separately assessing awareness for upper limb (eight questions on bimanual tasks) and lower limb (four questions on bipedal tasks) impairment. Patients are asked to use a 4-point visual analogue scale to estimate their current ability to execute a series of bimanual or bipedal actions that are presented in visual form by means of drawings. The patients’ scores are then compared to the scores attributed by the caregiver, and the difference between the two scores represents a measure of the severity of the AHP (see Table 1). Control items (unimanual actions and impossible actions) are also included in the test to check for the eventuality of patients giving implausible responses. In this way, the authors found evidence that AHP is underestimated in left hemisphere-damaged (LBD) patients when verbal interviews are used since 40% of their LBD sample (n. 33 in total) presented with signs of AHP [37].

As knowledge of the syndrome increased, new issues relating to AHP emerged, together with the idea that AHP, rather than being a unitary phenomenon, is instead the result of a combination of different factors [38].

Along with interviews, behavioural tasks were also introduced in AHP assessments, with patients being asked to perform certain actions and then provide a post-execution judgement of their performance [19,38]. In a test relating to the estimation of current ability in bilateral tasks [38], patients were asked to judge their proficiency when performing certain actions (“In your present state, how well, compared with your normal ability, can you … e.g., tie a knot? If you can do it as well as usual, say ‘ten’. If you cannot do it at all, say ‘nought’). When overestimations occurred (as compared to the judgement of the examiner), patients were asked to perform the action; then, the same questions were asked again. Furthermore, questions were asked from a third-person perspective (e.g., “If I were in your present state, how well would I be able to …, compared with my usual ability? If I could do it as well as usual, say ‘ten’. If I could not do it at all, say ‘nought’).

Thanks to the results of these approaches, the multifaceted nature of anosognosia has been acknowledged both in the literature and in clinical practice, with data indicating dissociations between implicit and explicit awareness [39]. Evidence of a further level of motor awareness, known as emergent motor awareness, has also been found [19,38].

With the aim of devising a comprehensive test which captures all of the different aspects of the syndrome, a multicentric, international group has recently published a new tool, the motor unawareness assessment (MUNA, [40]). Using existing studies as a guide, the MUNA, a 40-item battery, was developed and administered to 131 stroke patients suffering from right hemisphere lesions. It specifically assesses general awareness regarding the patient’s medical history and the reason(s) for their hospitalisation; awareness of upper and lower limb motor deficits; mentalisation (i.e., asking patients to consider a third-person perspective of their illness); confrontation tasks (i.e., asking them to perform specific movements); the patients’ capacity to anticipate their future clinical conditions and make predictions about recovery (i.e., anticipatory awareness); implicit awareness (i.e., analysing the patients’ behaviour during specific actions) and awareness of deficits in daily life activities.

The battery also includes a series of questions which investigate the symptoms which are often associated with AHP, including asomatognosia or a “disturbed sensation of limb ownership” (DSO, [11,41]), illusory movements [36], objectivation/personification [31], alien hand sensations [31] and emotional reactions toward the upper paralysed limb [31,38]. A principal component analysis identified five main factors (i.e., explicit motor awareness, implicit motor awareness, impaired sense of ownership, agency and illusory movement and emotional reactions), and a receiver operating characteristics (ROC) analysis was used to determine diagnostic cut-offs for the total scores, the motor awareness factor and all of the other components.

**Table 1:** Instruments for the assessment of the motor and non-motor dimensions of AHP. The table reports both the validated and non-validated tests which are used in clinical practice or suggested by research on AHP.

Authors	Methods	Object of the Assessment	Score Range/Cut-Off
Bisiach et al., 1986 [30]	Interview	From a general question about current clinical conditions toward more specific questions on motor deficits and request to move the upper or lower limb	0–3
Berti et al., 1996 [32]	Interview/movement request/ Capacity to perform daily life activities	From a general question toward specific ones Confrontation motor task Judgement on daily life actions	0–2 0–10
Feinberg et al., 2000 [36]	Interviews/ Questions from a third-person perspective	Specific questions Third-person perspective Confrontation tasks	0–1 0–10
Marcel et al., 2004 [38]	Interview/ Questions from a third person perspective/ Pre–post-action judgement–Estimation of current ability on bilateral tasks	Specific questions Third-person perspective Anticipatory awareness	≥5 (patient–examiner discrepancy)
Della Sala et al., 2009 [14]	Visual task with judgments on one’s own ability to perform actions (validated battery)	Awareness of daily life activities	<3.8 = Aware 3.8–8 = Mild AHP 8.1–16 = Moderate AHP >16 = Severe AHP
Moro et al., 2021 [40]	Interview/ Movement request/ Pre–post-interview/ Third-person perspective/ Capacity to perform daily life activities	Explicit motor awareness, implicit motor awareness, impaired sense of ownership, impaired sense of agency and illusory movement Emotional reactions	Cut-offs Total score: ≥27 Explicit awareness: ≥11 Implicit awareness: ≥0.75 Sense of ownership: ≥3 Sense agency: ≥2 Emotional reactions: ≥6 Robust score: ≥13
Cocchini et al., 2018 [13]	Errand choice test	Judging the difficulty of several motor tasks	318
Garbarini et al., 2012 [42]	Circle–line drawing task	Effects of the paralysed hand on healthy side motor planning	Analysis of the bimanual coupling effect
Cocchini et al., 2022 [35]	Vicarious motor task	Motor anticipation, sense of agency and sense of ownership for paralysed and healthy hands	VAS scores
Pacella et al., 2022 [19]	Action time estimation	Judging action durations	Temporal judgments of actions
Ramachandran and Rogers-Ramachandran, 1996 [34]	Fake injection deception (applicable during a normal drip)	Denial of paralysis	Report of the absence of movement
Besharati et al., 2014 [43]	Emotional negative feedback	Emotional modulation	Pre–post-awareness scores
D’Imperio et al., 2017 [22]	Potentially “dangerous” situations	Emotional modulation/anticipatory awareness	Pre–during action attempt and post-awareness scores
Besharati et al., 2016; 2024 [44,45]	Others’ perspective in visuo-spatial and verbal stories	Theory of mind	Spontaneous and multi-choice responses

## 3. Non-Motor Features of Anosognosia for Hemiplegia

### 3.1. Motor Imagery

Although AHP is defined as a specific disorder relating to the awareness of one’s own motor abilities, several experimental and neuroanatomical studies [19,26] have shown that other dimensions may contribute to the symptoms. From a clinical point of view, these “side” aspects need to be taken into consideration, especially during the assessment of patients and when determining rehabilitation strategies.

When the first explicative hypotheses regarding AHP were proposed, a debate emerged about whether the syndrome was due to a deficit in motor intention [46] or in motor monitoring [27], while less attention was given to aspects related to motor imagery.

Consequently, only in recent years have tools been devised to assess these dimensions. The errand choice test (ECT [13]) investigated the capacity of patients to judge the difficulty of several motor tasks, rather than estimating their own ability to perform them. Patients were presented with pairs of drawings depicting everyday tasks. In each trial, one figure represents a uni-manual task (e.g., “Comb your hair”) and a bimanual task (e.g., “Fold a sheet of paper in half”), and patients were asked to decide which of these they would find easier to perform in their current condition. When asked to judge the complexity of actions, patients performed like the healthy controls. However, when they reported on the difficulty they would have in executing the task, 31% of the brain-damaged patients (out of 73) displayed distorted perception. Furthermore, no differences between left hemisphere- and right hemisphere-damaged patients were found, even after checking for the patients’ ability to recognise and understand actions in order to exclude any potential confounding factors (e.g., limb apraxia [6]). Dissociations were found in some patients between their performance in the VATA-M and the ECT, confirming that a lack of awareness may occur for different reasons, and the related manifestations may not be identified correctly with only one method of assessment [13].

Bimanual paradigms have been used to examine whether, in cases of AHP, the motor programmes directed towards (but not executed by) the affected hand impact the trajectories of the movements of the non-affected healthy hand. Garbarini and colleagues [42] asked AHP patients to simultaneously draw lines with their unaffected hand and circles with their paralysed hand (i.e., circle–line drawing task; [47]). Of course, since this was only possible using their healthy hand, the movement of the paralysed hand was merely imagined. Nevertheless, the results of the task confirmed that the imagery of actions is spared, and intact spatial coupling effects were evident in these patients; indeed, the healthy hand produced an oval-shaped trajectory of lines due to the interference from the other hand which was merely imagining drawing circles.

Moreover, there is a confirmation of the importance of also assessing the behaviour of the healthy side of the body in a recent study by Cocchini et al. [35] in which the vicarious motor task [48,49] was administered to assess motor anticipation, sense of agency and sense of ownership in right hemisphere-damaged patients. The patients were asked to look in a mirror in front of them in which they could see the examiner’s hand performing various movements. The examiner was actually hidden behind the patient, and only his/her hand was visible in a position that was congruent with that of the patient, thus creating an illusory sense of embodiment of the examiner’s hand. The actions were either congruent or incongruent with a preceding verbal command given through headphones. The AHP patients displayed distortions in their sense of agency in the case of the incongruent action condition, for both the paralysed and the healthy hand.

Although normative values are not available for these last two tasks, they are in any case particularly useful from a clinical point of view in terms of being able to assess the potential effects of awareness disorders on the motricity of the healthy side of the body.

Finally, along with the abovementioned aspects related to motor imagery, the temporal dimensions of actions may also be impaired in AHP [50,51]. The action time estimation task (ATE; [51]) presents patients with a series of videos, which show the same actions being executed with different durations. Patients are asked to judge the duration of the action, and the effects of action embodiment in AHP are recorded. The data resulting from the experiment carried out by Pacella and collaborators [51] showed that embodiment modulates the estimation of the duration of actions in right hemisphere-damaged patients (RBD and AHP), thus confirming a facilitatory effect of embodiment in self-awareness.

### 3.2. Emotional Dimensions

The hypothesis that emotional reactions may contribute to AHP has been discussed since the 1930s [52,53] but has gained more weight with Weinstein and Kahn [54] and has been further strengthened by seminal studies on the differences in emotional behaviour as a consequence of the hemispheric side of the lesion [55]. While left hemisphere-damaged patients often show depressive (even catastrophic) emotional reactions to their clinical conditions, people with right hemisphere lesions tend to manifest indifference or minimise the severity of their paralysis and even joke about it [55]. This has been interpreted as the incapacity of AHP patients to face up to their deficits and the profoundly adverse changes in their lives. It may also represent a resistance to (or even a repression of) accepting the environmental and bodily signals relating to their inability to move a part of their body [25].

The debate on whether this is due to denial/refusal mechanisms or to difficulties in emotion regulation functions is still open [25], but it is undeniable that in clinical practice, patients who seem to completely reject the idea of being paralysed are common. While recognising other disorders (e.g., pain, problems with vision or internal disorders), they deny their motor deficits, sometimes using implausible excuses. However, fluctuations in patients’ responses, along with behaviours that show some implicit knowledge of their paralysis, might be signals of an awareness that is not being openly expressed.

Although tailor made, standardised tools for the assessment of denial are not currently available, so taking these aspects into account during an assessment is crucial. Information can be collected during the clinical interview [25,56] or by means of behavioural tasks derived from research. In a seminal study carried out by Ramachandran and Rogers-Ramachandran [34], an AHP patient, after a clinical interview, was subjected to a deception. He was informed that the doctor would inject his left arm with an anaesthetic as part of the neurological examination. The injection was in fact administered, but the syringe contained a saline solution that did not have any effect on the motricity of his arm. The doctor also said that, as an effect of the injection, the arm would be temporarily paralysed for a few minutes. After the injection, the patient reported that his left arm “seemed to want to do nothing” and was not moving. The same procedure, when administered to his right arm, did not deceive the patient.

Another way to assess whether emotional induction can modulate AHP relies on emotional feedback, either positive by using phrases such as “Well done” and “Your answer was very quick” or negative, such as “That is incorrect” or “You are not doing well in this task” [43]. This feedback induces emotional reactions in the patient. Experimental results showed that only negative emotion induction resulted in a significant improvement in motor awareness in the AHP patients as compared to the controls, while no effects were found with positive emotion induction. Similar results emerged from an error-based training task, in which the awareness of some patients improved when they were faced with their failure to perform an action (for emotional aspects [22]).

Finally, the contribution of emotional aspects to awareness can be evaluated with a behavioural approach based on which motor task the patient chooses. An action such as getting up from the wheelchair or washing an object in a sink may be judged to be possible or impossible by a patient, according to their situation or to the action itself. In this way, a patient who declares that they are able to get up and walk may then say that this would in fact be very difficult for them when they are faced with a flight of stairs. Similarly, a patient who declares that they can wash a spoon using two hands could at the same time say it would be difficult for them to clean a butcher’s knife because it is a potentially dangerous object [22]. In this way, the clinician can use very simple tasks to evaluate potential modulatory strategies aimed at improving the patient’s awareness.

### 3.3. Social Cognition in Anosognosia for Hemiplegia

Social cognition can modulate AHP symptoms [20,38]. There are patients who become aware of their deficits when asked to look at themselves from another perspective (i.e., “The doctor says you cannot move your leg, what do you think?”; “If I was in your current conditions, would I be able to move my arm?”; [38]) or when they look at themselves in a video [21,57].

Recently, Besharati and colleagues [44] devised a task in which both AHP patients and aware patients were requested to take the perspective of another individual in both visuo-spatial tasks and verbal tests of social cognition in which they were asked to make inferences about the mental state of other people (Theory of Mind, ToM). In this way, they demonstrated that AHP patients do not have deficits in visuo-spatial perspective taking as compared to right hemisphere-damaged patients without AHP. In contrast, they found selective deficits in AHP patients when they were asked to take on the perspective of another person and then verbally make inferences about their mental state [21]. Furthermore, these studies indicated that anosognosia and such ToM impairments are not merely correlated from a clinical point of view, but rather that AHP patients find it particularly difficult to apply social cognitive skills to paralysis-related content, especially when it is associated with their own condition [45]. An evaluation of these cognitive aspects, whether by means of interviews [38,40] or behavioural tasks [21,22], is thus to be deemed necessary in the assessment of AHP patients. 

## 4. Anosognosia and Concurrent Disorders

**Figure 1:**
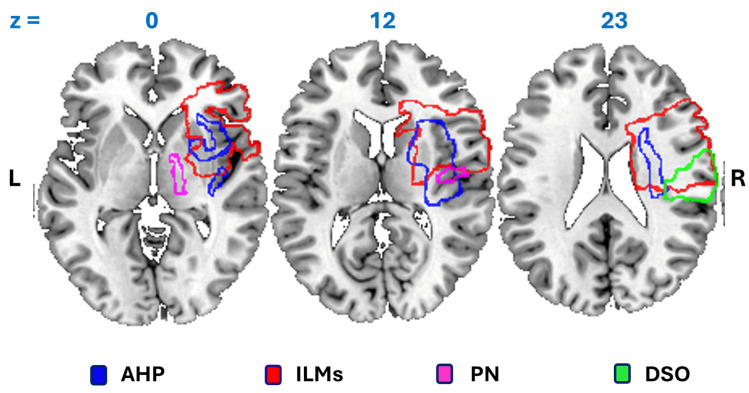
Partial overlapping of the neuroanatomical correlates with AHP and other frequently concurrent disorders. AHP—anosognosia for hemiplegia in blue; ILMs—illusory movements in red; PN—personal neglect in purple; DSO—disturbed sense of body ownership in green. Numbers refer to the Z-axis in the MNI coordinates. L: left; R: right. Adapted from Beccherle et al. [61]. Dorsal and ventral fronto-parietal attentional networks [67], with the involvement of the superior longitudinal fasciculus, are not reported.

As discussed above, the symptoms of AHP may be expressed in several clinical forms, which makes diagnosis problematic and challenging, particularly in the presence of mild disorders that often remain undiagnosed [14]. Further potential difficulties may arise from the frequent presence of concomitant symptoms, such as fluctuations in attention [58,59], visuo-spatial and personal neglect [20], disorders in body representations [58,60] or illusory, involuntary movements [36,61] (Figure 1). In depth, neuropsychological and neuroimaging examinations [62,63,64,65,66] have demonstrated dissociations between these clinical conditions, and thus, differential diagnoses are crucially important since they allow the patients’ clinical profile to be correctly and precisely identified.

### 4.1. Vigilance/Alertness

In the more acute phases, AHP patients are often disoriented and are not sufficiently vigilant to give consistent responses, and at times, they confabulate or appear sleepy. These symptoms fluctuate and may be part of the normal clinical course (due to the involvement of fronto-parietal attentional networks [67]), but sometimes, they are directly associated with substantial changes in the patient’s emotional state resulting from his/her motor deficits [43]. In these cases, sleepiness or confabulatory responses might represent a sort of avoidance, namely a strategy used to avoid specific requests that the patient knows, in some way, that they are not able to respond to [22]. Only a clinician has the expertise to understand when these responses are to be considered as an index of general reduced vigilance, which may also be associated with non-restorative sleep, hypoglycaemia, dehydration, a change in the pharmaceutical treatment or fatigue. However, the systematic recording of the patients’ reactions to conversations linked to their paralysis is useful, as it helps the clinician identify the presence of these reactions, which reveal avoidance or defence mechanisms. To record the patient’s behaviours and see if they can be indicators of avoidance, the examiner can use simple recording sheets. An example is shown in Table 2.

### 4.2. Spatial Neglect

AHP was initially described as a disorder which is closely related to (or even secondary to) spatial neglect. There are essentially two reasons for this. The first regards the overlapping of the lesions associated with the two clinical conditions. This is frequently reported, in particular in the right temporo-parietal junction, the superior and middle temporal gyri and the right insula [15,68,69,70,71,72]. The second reason originates from early studies using experimental manipulations to modulate spatial neglect (e.g., vestibular stimulation [73,74,75]), which showed a parallel recovery path for the two syndromes. More recently, in-depth investigations have shown that the responses to interventions are indeed different, not only with respect to the two clinical conditions but also among individual patients, probably due to the multifaceted nature of both syndromes [76]. Dissociations have been documented, with spatial neglect present without AHP and AHP present without neglect [30,77,78]. This, in addition to a number of controlled neuroimaging investigations [19,27,28], demonstrates that the two conditions are independent.

Cases of personal neglect are less easy to classify. Patients with this condition behave as if the contralesional part of their body does not exist, and thus, for example, might appear with only half of their face shaved or made up, their hair only combed on the ipsilesional side or with their glasses misplaced on the contralesional side of their head [64,65].

In clinical practice, a failure to directly attend to contralesional body parts (neglect) or a failure to acknowledge or recognise contralesional paralysis (anosognosia) may appear very similar. Furthermore, some of the tasks used to assess personal neglect do not specifically provide an in-depth evaluation of the syndrome (e.g., the one-item test does not differentiate between personal neglect and asomatognosia or directional hypokinesia [79]). This may lead to mistakes in the diagnosis.

However, the two syndromes are conceptually different, since while AHP represents a deficit in motor awareness [19], personal neglect relates to a lack of attention towards the contralesional body part.

Along with the use of standardised tests, a simple way to distinguish the two syndromes is by forcing the patient to pay attention to their contralesional body part (e.g., their hand) when positioned in the ipsilesional space [78]. Patients suffering from personal neglect (but not AHP) will find their hand, recognise it as their own hand (if asomatognosia is not present, see below) and even admit their motor deficits. In contrast, AHP patients will continue to deny their paralysis and also try to perform movements that are in practice impossible for them.

### 4.3. Body Ownership Disorders and Illusory Movements

“Disturbed sensations of limb ownership” (DSO [11]) is a classification used to indicate all abnormal feelings and beliefs regarding the existence and ownership of one’s own limbs. This includes asomatognosia, that is, the sensation or feeling that one’s body or a body part does not belong or is not present (e.g., abnormalities in the experience of existing, in visual self-recognition or in the sense of body parts belonging, with the body part sometimes being attributed to another person [41]), and somatoparaphrenia, in which there are illusional, confabulatory or delusional ideas of disownership (e.g., supernumerary limbs, objectivation and personification [41,58]).

Clinical dissociations between AHP and body ownership disturbances have been described since Gerstmann’s seminal paper [80] on the topic. The critical difference seems to be that, while AHP affects patients’ awareness of their actions, a right hemisphere stroke can also cause abnormalities in the sense of body ownership, regardless of whether the limb is paralysed or not. AHP patients without DSO will recognise their own arm and declare that they are able to move it. In contrast, DSO patients without AHP acknowledge that the limb is paralysed but also declare that the limb does not belong to them.

Nowadays, clinicians have specific tools to assess these distinct disorders, and these have been administered to large groups of patients, providing normative values which are useful in terms of the identification of all these different symptoms (see, for example, the Affected Limb Perception Questionnaire (ALPQ) [60] and the Disturbed Sense of Limb Ownership Questionnaire [66]). However, these tools must always be integrated with clinical observation, which plays a crucial role, especially in the treatment of these patients. In fact, it may happen that, although patients respond (or learn to respond) correctly during interviews, hidden symptoms emerge when their cognitive control is reduced because they are engaged in other activities (for example during motor rehabilitation).

Finally, the presence of illusory limb movements (ILMs) seems in some way to represent the opposite of AHP. Patients with ILMs report having the illusion of involuntary, uncontrollable movements executed by the contralesional upper limb [61,81], sometimes mirroring the healthy limb.

Significantly, the movements do not actually occur, unlike in the case of anarchic hand syndrome [14,82]. For this reason, ILMs have been typically considered to be associated with anosognosia for hemiplegia (AHP) [83] or with DSO [36,41]. However, the interactions between these clinical conditions remain underexplored and, given their rarity, have mainly been studied in single cases. Indeed, a patient has recently been described as reporting ILMs without any relevant signs of AHP, DSO, somatosensory deficits or contralesional spatial neglect [61]. The neuroanatomical investigation showed that this patient’s lesion was more anterior, lateral and dorsal than in cases of AHP, and it did not involve the medial networks which are typically associated with AHP and DSO (i.e., the middle cingulum, the inferior parietal lobule, the supplementary motor area, the hippocampal complex/amygdala and the precuneus or the insula [19,84,85]). Thus, the hypothesis proposed by the authors of this paper is that ILMs may arise from a disconnection between the motor and sensory systems that, in normal conditions, are integrated during both action execution and states of rest [61].

## 5. Limitations and Conclusions

The main limitation of this study regards the process of the literature review on AHP assessments, which did not follow a systematic procedure. In fact, our aim was not to provide a systematic review but rather to discuss the evolution of assessment tools. Another limitation is the lack of comparison between the instruments for the assessment of AHP and those that are used in clinical contexts for anosognosia in other domains.

There is general agreement regarding the idea that AHP is a multi-factorial syndrome with dynamic relations between different aspects, as well as interactions between bottom-up and top-down processes [2,26,86]. As a consequence, any assessment of AHP needs to take all these different facets into consideration. Nowadays, the neuropsychological tools which are available can help clinicians with the diagnosis, but it is still crucial that clinical manifestations are closely observed by going more deeply than in formal examinations.

The complexity of the syndrome requires an integrated, interdisciplinary and embodied approach to the diagnosis [4,87] that values the information gathered from both the clinical team and family members, and it should also take the role of social and environmental influences into account.

It is also worth noting that the assessment procedures will differ according to the aim. When the aim of the evaluation is exclusively diagnostic, the neuropsychologist may be faced with time constraints, which means that the time available to collect all the relevant information is significantly reduced. For this reason, a standardised, comprehensive battery of tests, including all the facets of AHP, is recommended. However, if the aim of the assessment regards rehabilitation and is carried out in order to determine the intervention strategies to be implemented, integrating different instruments (e.g., verbal and non-verbal requests or behavioural tasks) and paying attention to differential diagnosis are fundamental. One advantage associated with this latter type of assessment is that patients can be observed more than once, namely at different times of the day and in different contexts. Complex clinical conditions require not only technical abilities but also the time to reflect and seek answers to the questions that may be more relevant and crucial to the patients in terms of their recovery.

## Figures and Tables

**Table 2:** Monitoring of the evolution of clinical symptoms over time. Legend to fill in the recording sheet +: presence of deficit; NT: not testable for clinical reasons, NP: symptom no longer present. Scores for clinical symptoms from 1 (severe deficit) to 5 (no deficit) are rated as follows: attention—scores from 1 (no attention) to 5 (complete attention); fluency—scores from 1 (mute) to 5 (loquacious); confabulation—scores from 1 (inconsistent language) to 5 (consistent language); avoidance—scores from 1 (very frequent) to 5 (not present at all); referred pain—scores from 1 (excessive complaints) to 5 (not reported); fatigability—scores from 1 (high degree) to 5 (absent); gaze deviation—L/R: presence of gaze deviation toward the left/right side; sleepiness: scores from 1 (high degree) to 5 (absent) (modified by D’Imperio et al. [59]).

	Days Since Lesion										
Symptoms		1	2	3	4	5	6	7	…	…	…
Neglect											
Attention											
Fluency											
Confabulations											
Avoidance											
Referred Pain											
Fatigability											
Gaze deviation											
Sleepiness											

## Data Availability

No new data were created or analysed in this study. Data sharing is not applicable to this article.

## Abbreviations

The following abbreviations are used in this manuscript:

AHP	Anosognosia for Hemiplegia
LBD	Left Brain Damage
RBD	Right Brain Damage
VATA-m	Visual Analogue Test for Anosognosia for motor impairment
MUNA	Motor Unawareness Assessment
DSO	Disturbed Sensation of limb Ownership
ECT	Errand Choice Test
ATE	Action Time Estimation task
ToM	Theory of Mind
ALPQ	Affected Limb Perception Questionnaire
ILMs	Illusory Limb Movements
